# Cryopreservation/transplantation of ovarian tissue and in vitro maturation of follicles and oocytes: Challenges for fertility preservation

**DOI:** 10.1186/1477-7827-6-47

**Published:** 2008-10-02

**Authors:** Alex C Varghese, Stefan S du Plessis, Tommaso Falcone, Ashok Agarwal

**Affiliations:** 1 Center for Reproductive Medicine, Glickman Urological and Kidney Institute and Department of Obstetrics and Gynecology and Women's Health Institute, Cleveland Clinic, Ohio, USA; 2 Division of Medical Physiology, University of Stellenbosch, Tygerberg, South Africa

## Abstract

Cryopreservation of ovarian tissue and in vitro follicle maturation are two emerging techniques for fertility preservation, especially in cancer patients. These treatment regimes are opening up more options and allow for more suitable choices to preserve fertility according to the patient's specific circumstances. If these technologies are to become widely accepted, they need to be safe, easy to perform and must obtain favorable results. The generation of healthy eggs with the normal genetic complement and the ability to develop into viable and healthy embryos requires tight regulation of oocyte development and maturation. Novel freezing techniques such as vitrification, along with whole ovary cryopreservation and three-dimensional follicle cultures, have shown favorable outcomes. The scope of this article is to take a comprehensively look at the challenges still faced in order for these novel technologies to be routinely employed with the aim of successful fertility preservation.

## Background

Despite the availability of several options for fertility preservation in patients experiencing premature ovarian failure (POF) due to chemo- or radiotherapy, embryo cryopreservation remains the only established method of preserving fertility, according to The Ethics Committee of the American Society for Reproductive Medicine (ASRM) [1,2]. This may not be the most appropriate strategy as most patients are not able to postpone cancer treatment to undergo a cycle of ovarian stimulation. Furthermore, the patient is required to be of pubertal age and to have a partner or make use of donor sperm, rendering this method not suitable to a large percentage of these patients.

The remaining options are either experimental or have not been fully evaluated and thus can not be proposed to patients at this time [3]. Cryopreservation/transplantation of ovarian tissue and *in vitro *maturation (IVM) of follicles/oocytes are two such emerging techniques. The aforementioned treatment regimes are opening up more alternatives and allow for more suitable choices to preserve fertility according to the patient's specific situation. For these technologies to be recognized and carried out routinely, they must be safe, easy to perform and deliver successful results. As these technologies are still quite novel, the goal of this article is to offer an in-depth look at the challenges that must be overcome with the cryopreserving and transplanting of ovarian tissue as well as the IVM of follicle and oocyte techniques to ultimately lead to successful fertility preservation.

## Cryopreservation and transplantation of ovarian tissue

Ovarian tissue cryopreservation and transplantation is still an experimental procedure for preserving fertility in woman faced with failure of reproductive functions. This technique allows for completion of medical therapy to treat the primary medical disorder with, for example, gonadotoxic drugs. Ovarian tissue can be harvested at laparoscopy or laparotomy by performing several ovarian biopsies, or a partial, uni- or bilateral oophorectomy and cryopreserved. The extirpated and cryopreserved ovarian tissue subsequently can be autotransplanted upon completion of the medical therapy and after the patient is in remission. It furthermore can serve as a source of follicles for IVM.

**Figure 1:**
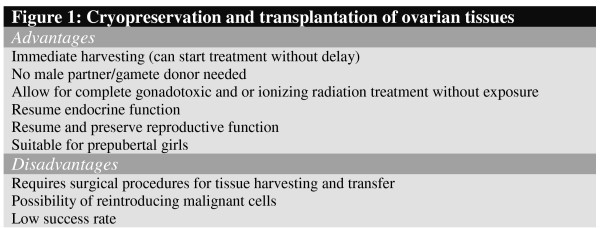
Cryopreservation and transplantation of ovarian tissues.

There are various advantages of ovarian tissue cryopreservation (see Figure 1). Firstly the tissue can be obtained without delay since there is no need for ovarian stimulation; while no partner is required for male gamete donation at the time of tissue harvesting. These procedures offer the advantages of allowing for resumption of reproductive and endocrine function (obviating the need for hormone replacement therapy) and avoiding immunosuppressant medications by performing autotransplantation. Ovarian tissue cryopreservation also may be a more viable alternative to cryopreservation of oocytes or embryos, particularly for pediatric cancer patients. The disadvantages of ovarian tissue cryopreservation are that it requires surgical procedures for tissue harvesting and transfer and there have been only a few pregnancies world-wide. It is also not an option for women with ovarian cancer as the risk of reintroducing malignant cells into cancer patients by autografting ovarian cortical fragments is one main concern. An alternative is isolation of intact Graafian follicles or pre-antral follicles from freeze-thawed ovarian tissue and re-transplantation. However, isolation of follicles by either mechanical or enzymatic means is cumbersome, especially from human ovaries having fibrous tissues. It is less clear whether cryopreserved ovarian tissue should be utilized in women who have an estrogen-dependent cancer.

## Cryopreservation techniques

Martinez-Madrid *et al*. described a cryopreservation protocol using a passive cooling device for an intact human ovary with its vascular pedicle. This protocol showed high survival rates of follicles (75.1%), small vessels and stroma, and a normal histological structure in all the ovarian components after thawing [4]. In a follow-up study, Martinez-Madrid *et al*. [5] compared fresh to cryopreserved-thawed whole human ovaries with their vascular pedicle, assessed according to ultrastructure and apoptosis. They detected no significant differences in DNA strand breaks by TUNEL, immunohistochemistry, or activation of active caspase-3 after thawing. Also, no discernible ultrastructural alterations were encountered in these frozen-thawed ovaries as assessed by transmission electron microscopy. These findings are further evidence that their present freeze-thawing protocol [4] does not cause significant injury to human ovaries and may be a suitable technique to cryopreserve whole ovaries until transplantation.

Recently, several research groups also have assessed intact ovarian tissue cryopreservation and transplantation efficiency in animal models. Bedaiwy *et al*. [6] have shown that an intact sheep ovary can survive cryopreservation-thawing insults with reasonable tissue viability. The grafts were perfused via the ovarian artery with the cryoprotective mixture using the Horizon^® ^Nxt Modular Infusion System (B. Braun Medical, Inc., Bethleham, Pa.) to maintain a flow rate at 1.3 mL/min with continuous replenishment of the reservoir. The researchers conclude that perfusion of the cryoprotectant through the vascular channel and autotransplantation of an intact, frozen-thawed ovary with microvascular anastomosis is technically feasible with good short-term results. In a further study Bedaiwy *et al*. [7] compared cryopreserved-thawed intact ovaries with ovarian cortical strips from tissue obtained from women undergoing bilateral oophorectomy. One of the harvested ovaries was sectioned and cryopreserved (by slow freezing) as ovarian cortical strips (1.0 × 1.0 × 5.0 mm), while the other ovary was cryopreserved intact with its vascular pedicle. After thawing 7 days later results were comparable thereby proving that using human intact ovaries, cryoperfusion and cryopreservation of the entire human ovary can be achieved with the maintenance of excellent viability of the superficial and the deeper tissues using a slow-freezing protocol. Cryopreservation injury was associated with neither significant alteration in the expression pattern of Bcl-2 and p53 proteins in the ovarian tissues nor significant follicular damage.

Based on the newly developed technology of directional freezing (multi-thermal gradient), which is said to freeze biological samples at a very low and accurate cooling rate [8] and control the ice crystal morphology, Arav *et al*. [9] studied the transplantation efficiency of frozen-thawed, intact sheep ovaries by artery and vein anastomosis. Whole sheep ovaries, comprising the follicles and blood vessels, survived cryopreservation, and ovarian activity was seen as early as 2 months after transplantation. In addition, magnetic resonance imaging has shown that blood vessels were intact and that normal blood flow to the transplant had resumed. Their study shows that immediate and long-term hormonal restoration and normal ovulation are possible after cryopreservation and transplantation of whole ovaries in sheep.

Imhof *et al*. [10] reported pregnancy and live birth by orthotopic microvascular re-anastomosis of whole frozen-thawed sheep ovaries. These authors used a slow freezing/rapid thawing method for the cryopreservation of the intact ovaries with vascular pedicles following a perfusion step with cryoprotectants. However, follicular loss was higher as revealed by histological examination (follicular survival rate ~4.3%). The authors re-emphasize the need for further refinements in cryopreservation regimens so as to increase the follicular survival rate.

## Transplantation options

To date various techniques for transplantation of ovarian cryopreserved tissue exist. Autotransplantation would be beneficial as it would avoid dealing with immunosuppressant treatments. The most advantageous option would be orthotopic autotransplantation as it might furthermore lead to natural conception, while heterotopic transplantation is another autotransplantation alternative. Both allotransplantation (same species) as well as xenografting into animals of a different species also provides further options and treatments.

Successful animal experiments were performed in the early 90's in which transplantation of cryopreserved-thawed ovarian cortical strips led to follicular survival and endocrine function, as well as pregnancy and delivery [11,12]. The first human pregnancy by cryopreserved ovarian cortex and transplantation was recently reported [13]. In 2005, Meirow *et al*. also reported a live birth after orthotopic autotransplantation of cryopreserved ovarian tissue in a patient with POF after chemotherapy [14].

Because of the limitations (ischemic injury, delays in neovascularization) of fresh and cryopreserved-thawed ovarian cortical strips, intact ovarian tissue grafting in conjunction with cryopreservation is thought to be potentially a better option to preserve fertility. Until now, autotransplantation procedures have been limited almost exclusively to non-vascular cortex segments grafted to either orthotopic or heterotopic locations. The disadvantage of non-vascular grafts is that they often sustain significant ischemic injury until tissue revascularization takes place [15]. The reduction of the ischemic time-interval after re-implantation is therefore crucial for the reproductive potential of the transplants. In general a large number of follicles (up to two-thirds) are lost shortly after transplantation of cryopreserved ovarian tissue. This loss is unlikely to be due to freezing and thawing, but more likely caused by post-transplantation ischemia. Israely *et al*. [16] have shown that a reduction in ischemic damage is possible by transplantation into granulation tissue. They have shown that transplantation into angiogenic granulation tissue, created during wound healing, shortened the ischemic period by 24 hours and significantly increased the pool of healthy primordial follicles and the perfused area of the transplanted grafts.

A vascular transplant has been suggested as the most logical approach compared with transplantation of ovarian cortical tissue. Despite the technical difficulty of the transplantation procedure, it grants immediate blood supply to the transplant, thus minimizing ischemic injury.

## Safety issues and future prospects

Current cryopreservation protocols appear to affect crucial interactions between the oocyte and granulosa cells, especially the crucial communications via transzonal processes containing filamentous actin (TZPs-Act). A study comparing the efficacy of cryoprotective agents like dimethyl sulfoxide (DMSO) and glycerol on gap junctional communications during ovarian tissue cryopreservation has shown that DMSO is superior to glycerol in maintaining a higher post-thaw TZPs-Act density because of its higher tissue permeation coefficient [17]. Both cryopreservation and cryoprotectant exposure have been shown to compromise the integrity of the granulosa-oocyte interface, suggesting that further optimization of cryopreservation protocols is still required to curtail the disruption of cellular communication routes in the ovary. Any disruptions in communication pathways required for the integration of oogenesis and folliculogenesis have been shown to result in either inappropriately timed or defective chromatin remodeling that leads to specific defects in meiotic competence [18]. One of the consequences of a loss in coordination is believed to be caused by the failure to appropriately regulate gene expression in the developing oocyte and/or the acquisition of meiotic competence. The need to sustain and coordinate these processes during normal ovarian cyclicity or during ovarian stimulation or any *in vivo/ex vivo *manipulations like ovarian tissue cryopreservation and transplantation is tantamount to the production of developmentally competent oocytes for use in assisted reproduction technologies.

Experimental evidences indicate that damage due to extracellular ice is the single most serious obstacle to the extension of cryopreservation techniques to multicellular systems [19]. The ice-free vitrification method of ovarian tissue preservation may prevent intravascular ice formation, which leads to failure of blood supply following grafting and vascular anastomosis. However, one major concern is the temperature gradient during the warming step that might induce damages in vitrified bulky samples like ovarian tissue and whole organs. The possibility of using electromagnetic heating is being studied for this purpose [20]. Several anti-freeze proteins and ice blockers have been shown to reduce the critical warming rate and thereby avoid the devitrification effect of some cryoprotectant solutions [21]. An important step in designing a vitrification procedure is to measure the penetration of the vitrification solutes throughout the system and assess the cell-volume response to the changes in osmolarity that will be caused by the added solutes [22]. Rahimi *et al*. [23] compared necrosis in human ovarian tissue after conventional slow freezing or vitrification and subsequent xenotranplantation in severe combined immunodeficiency (SCID) mice. There was no significant difference in necrosis between the groups.

Development of new cryo-chambers, vascular perfusion systems and warming devices to avoid de-vitrification or recrystalization, as well as improving cryopreservation protocols for the intact ovary, are considered vital directions in ongoing research to make the transplantation of an entire ovary a realistic objective [22,24]. A novel method of direct cover vitrification has been shown to increase follicular viability and pregnancy capability in mice ovarian tissue [25]. It uses less concentrated cryoprotectant and maximizes the cooling rate by utilizing direct cover with liquid nitrogen. Migishiga *et al*. [26] developed a different protocol for the cryopreservation of whole ovaries by vitrification. DAP 213 (2 M DMSO, 1 M acetamide and 3 M propylene glycol) was used as cryoprotectant after a prior equilibration in 1 M DMSO at 5°C. Even though various strategies have been reported in the literature for freezing of ovaries, the oocyte competence and developmental potential after re-transplantation seems low. Further refinement in technology, especially ice-free vitrification, is warranted. It is suggested that the cryopreservation procedure may have some negative effects even on the residual recipient's ovary as well as on the ovarian graft and that the cryoprotectant remaining in the transplanted graft might be one of the factors causing these deleterious effects [26]. Based on the reports showing improvements during oocyte cryopreservation using higher sucrose concentration, Bianchi *et al*. [27] studied the effect of different sucrose concentration during ovarian tissue freezing. Human oocytes were cryopreserved using a modified slow-cooling protocol involving 1.5 mol/l propane-1,2-diol (PrOH) and 0.2 mol/l sucrose during dehydration, while rehydration was conducted applying decreasing concentrations of PrOH and 0.3 mol/l sucrose. Increased oocyte survival rates were achieved by these moderately high sucrose concentrations in the freezing and thawing solutions. This also ensures elevated success rates in terms of fertilization, embryo development and clinical outcome. The use of 0.3 mol/l sucrose showed a smaller percentage of damaged germ cells than 0.2 mol/l sucrose, and therefore was less detrimental to the thawed ovarian tissue based on their electron microscopic histological observations.

According to the Practice Committee of the ASRM [28], ethical and safety issues are to be considered at different levels when applying ovarian tissue cryopreservation and transplantation in patients [29] as this procedure can lead to reintroducing malignant cells, as mentioned previously. The safety of this procedure can be compromised by the transmission of lymphoma via grafting of ovarian tissue from diseased donor mice to healthy recipients [30]. Intensive collaboration between oncologists and fertility-preservation specialists is required for correct estimation of risk for each patient. Though in its infancy, xenografting [31] or *in vitro *follicle culture could be future solutions for individuals at risk of cancer re-transmission. Blastocyst development and birth of pups derived from vitrified preantral follicles and ovarian tissue have been reported recently [32,33]. Kagawa *et al*. [34] also reported the birth of 10 healthy mouse pups derived from oocytes obtained from preantral follicles using adult ovarian tissue vitrification and allotransplantation in SCID mice. An apoptotic and necrotic pathway of cellular reduction has been reported in cryopreserved mouse ovaries. It has been reported that cryopreserved sheep preantral follicles underwent growth *in vitro *but that freezing/thawing specifically affected gap junctional permeability and impaired the progression of regulative processes; such as the acquisition of a specific oocyte chromatin configuration [35].

Today's technological advances allow for a complete genetic assessment of cells and tissues through microarray-based global gene expression patterns and MALDI-TOF- based proteomic studies. Future research should utilize these novel and potential technologies to unravel the molecular information of germ cells as well as somatic cells in ovarian tissues post-cryopreservation and -transplantation to nude mice that can support the growth of such tissues in a synchronous and coordinated way. Kim *et al*. [36] have studied the proteome of cryopreserved bovine ovarian tissue and shown alterations in protein profiling and expression in ovarian grafts after transplantation. The most significant of the identified proteins after transplantation were those related to tissue survival and metabolism, such as actin and laminin, and antioxidant properties (glutathione S-transferase). Glutathione-S-transferase is an enzyme that is usually upregulated in oxidative stress and mRNA; it also has been shown to be upregulated in mouse embryos under cold stress response [37]. Among the slow-freeze and vitrification groups, the change in protein profile of the vitrified and transplanted ovarian tissue was more consistent with that of the fresh controls [36] and, therefore, possibly holds the key for optimizing this technique. The latest technical developments such as laser capture microdissection can be used to obtain pure cancer cells from fresh-frozen cancer tissue and the surrounding environment, thus providing an accurate snapshot of the tumor and its microenvironment *in vivo*. Silasi *et al*. [38] reported a new approach to isolate pure cancer cell populations and evaluate protein expression. The process includes immunocytochemistry, laser microdissection, and western blot analysis. Using this technique, it is suggested that proteins such as X-linked inhibitor of apoptosis protein and Fas ligand could be detected with as little as 1000 cells. These approaches might be useful in detection of any cancer cells in frozen-thawed ovaries prior to transplantation back to the patient.

Research and development of technology to cryopreserve whole organs, as well as surgical techniques for the autotransplantation of an entire ovary with its vascular pedicle, are being intensely pursued by several research groups. This could lead to the transplantation of intact ovaries with microvascular anastomosis carried out to restore immediate vascularization and minimize post-transplantation ischemia, responsible for the reduction in follicular density.

According to Bedaiwy [3] the ultimate challenge to achieve a successful ovarian implant is for the ovarian fragment or intact ovary to 1) survive the freeze-thaw trauma with minimal damage to the follicular structure, 2) establish blood supply immediately upon transplantation, 3) regain reproductive and endocrine function and 4) survive for a considerable time.

## In vitro growth of primordial follicles and in vitro maturation of oocytes from antral follicles

As a consequence of radiation and chemotherapy, many females suffer from POF and premature menopause. Cryopreservation of mature oocytes has shown limited success, which means that very few options for putative fertility restoration are available to these patients [39]. In fetal and adult human ovaries, most of the follicles remain primordial [40] and, therefore, immature oocytes are regularly available and would be the easiest and most abundant source of female gametes. However, they still must grow and undergo maturation in order to become fertilizable, and this has to be obtained *in vitro*.

**Figure 2:**
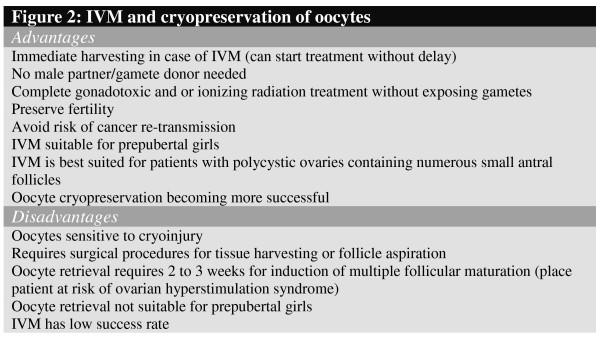
IVM and cryopreservation of oocytes.

Keeping pace with efforts to refine *in vitro *fertilization (IVF) techniques, active research is continuing in the area of folliculogenesis and *in vitro *oocyte maturation. Progress in this field will benefit patients who have stored ovarian tissues prior to debilitating cancer treatments. This also may allow patients a realistic chance of conceiving a child at an advanced age (e.g., career-orientated women who wish to postpone childbearing) if ovarian material was harvested from the ovary at an early age and stored. Healthy immature oocytes can be retrieved at a later stage and matured *in vitro *for IVF. Moreover, compared to conventional IVF, the advantages of this approach include no hormonal down-regulation or hormone injections in regularly cycling women; minimal stimulation with follicle stimulating hormone (FSH) in women with polycystic ovaries (PCO); no ovulation injection, thereby minimizing any side effects and discomforts; and reduction in treatment span and interference with daily life combined with less emotional stress. IVM of primordial follicles also would avoid the possible risk of re-transmission of some cancers, such as hematological malignancies [41] and breast cancer [42], by ovarian grafts as oocytes do not contain cancerous cells. However, the growth and IVM of primordial follicles remains a major challenge for reproductive biologists (Figure 2).

## In vitro follicle culture

The ovarian physiology of mice and knockout mice models has been studied extensively, and *in vitro *culturing of distinct stages of preantral and antral follicles has provided much information on somatic and germ cells interactions during growth and maturation [43,44]. Only a single offspring has been produced so far in the mouse model by IVF after the growth and maturation of primordial follicles *in vitro *[45,46]. A normal expression pattern of growth differentiation factor (GDF-9) and anti-Müllerian hormone has been reported in human follicles cultured *in vitro *after freeze-thaw cycles [47]. This study further point out that a defined serum free medium could support transition from resting to growing follicles leading to the development of secondary follicles during the *in vitro *follicle culture of frozen thawed human ovarian tissues.

Primordial follicles can be isolated from either fresh or cryopreserved ovarian tissues. These follicles typically contain a small oocyte arrested at the diplotene stage of the meiosis and surrounded by a single layer of flattened pregranulosa cells and a basement membrane. Primordial follicles are located in a narrow cortical band in primate ovaries under a poorly vascularized tunica albuginea. A cortical biopsy of ~1 mm thick can yield sufficient follicle-rich region without much bleeding as prominent blood vessels lie deep in the medulla. Isolation of primordial follicles involves either mechanical or enzymatic treatment. Avoiding damage to the basement membrane and other intra- follicular compartments is a major challenge during the harvesting of these follicles, and further *in vitro *developmental potential largely depends on a successful harvesting procedure. Follicles also can be grown in organ explants until they have grown to a stage at which they can be isolated as granulosa-oocyte complexes and transferred to either collagen membrane or three-dimensional culture systems. Otherwise, cultured organ explants can be transplanted to host animals to complete the follicular development. As recently demonstrated [48], liberase treatment allows the isolation of highly viable follicles with an unaltered morphology and ultra structure. This enzyme preparation is said to be a promising alternative to collagenase preparations for the reproducible isolation of intact primordial follicles for culture and grafting purposes. The same authors recently have shown that isolated human follicles are able to survive and grow after xenografting [49], as they observed well-structured, stroma-like tissue of human origin around the isolated follicles one week after grafting.

For domestic animals and humans, the technology is still far from successful; there are only a few reports of limited success using *in vitro *culture of large preantral follicles that have progressed further developmentally [50,51]. The large, preantral follicles contain oocytes that are almost fully grown; however, the meiotic competence of these follicles remains low, indicating that the culture methods require optimization. As the store of female gametes is maintained within the primordial follicles, the real challenge is to establish the culturing of primordial follicles. Unraveling the regulation of the transition from primordial to primary follicle is the key to accessing an almost unlimited source of oocytes for biomedical use.

Each species has its own specific developmental timeline. In rodents (mice, rats), the time span between initiation of follicle growth and formation of the antral cavity is a few weeks and in large domestic animals it takes several months. During the preantral growth phase, the oocyte grows rapidly and reaches almost its maximum volume when the first accumulation of fluid is observed within the granulosa cell mass. In humans, Gougeon [52] estimated that the growth phase from primordial to primary follicle in humans takes > 120 days. Once in the growing pool, the follicle requires 65 days to reach the early antral phase (follicle of 2–5 mm diameter). At this point it becomes dependent on gonadotrophins for further growth.

Factors regulating ovarian follicle growth, death and recruitment still need to be elucidated fully. Hence, choosing an artificial culture system will always mean a compromise. When the aim is to mimic normal physiology and to culture the follicles as multilayered spheres, the supply of essential nutritional and physical factors to the inner centre of the structure becomes critical [53]. One potential limitation of the conventional culture systems is the disruption of follicle architecture that can occur when follicles are cultured on a two-dimensional substrate [54]. The change in follicle morphology may alter the paracrine signaling that is critical to follicle maturation because the altered cell-cell orientation could result in diffusion of paracrine signals away from the target cells. The culture environments and media compositions tested to date appear incapable of creating the right environment for inducing growth changes that are compatible with physiological growth patterns. It was found that FSH and insulin [55,56] promote the growth and survival of the follicles as well as GDF- 9 [57]. Growth factors such as cGMP also showed improvement in follicle survival [58]. The optimal culture media for growth promotion may need to be very rich in supplements and be species-specific. Using different compositions of media sequentially for every follicular stage also may be necessary until a mature, healthy and fertilizable human oocyte is obtained [59].

The recent application of tissue engineering technologies to follicle culture has opened up new insights into follicle physiology *in vitro *and yielded favorable results [60]. The novel three-dimensional culture system, in which individual immature mouse granulosa-oocyte complexes or intact follicles are encapsulated within alginate beads, is one such system [61,62]. Alginate is a linear polysaccharide derived from algae and composed of repeating units of β-d-mannuronic acid and α-L-glucuronic acid and gels by ionic cross linking of the glucuronic residues [63]. In this system, the alginate matrix provides a mechanical support for the follicle as it increases in size, allowing examination of the role of various factors in follicle growth while maintaining an *in vivo*-like morphology. Additionally, encapsulating the follicle within a three-dimensional matrix allows for studying the interactions of the outer layers of somatic cells and insoluble factors, such as the extracellular matrix, which direct follicle growth and maturation. Culture systems like the three-dimensional one that can be tailored to the developmental stage of the follicle will be especially critical for translation to human follicles, which require several months to mature [64].

The developmental transition of primordial (oocytes in late diplotene stage) to primary follicles is characterized by a cuboidal appearance of the granulosa cells (a process of 120 days) from a flattened nature and initial growth of the nucleus. These changes, however, occur within a few days *in vitro *[65]. The regulatory mechanisms that coordinate oocyte growth and cell division in the surrounding somatic cells are largely unknown. The encouraging results with three-dimensional culture of murine preantral follicles along with extra-cellular matrix and activin-A emphasize that such an environment provides a sufficient *in vitro *milieu for follicle growth and survival [66].

There is ample opportunity for improvement in this area as the technical requirements and the selection of essential biochemical determinants for the preantral stages of follicle development need to be characterized precisely. Mapping these stage-related biochemical determinants of follicle growth requires investigation of follicles at well-characterized growth stages using new, powerful molecular biology techniques such as gene chip technology, proteomics and metabolomics. Interaction among professionals in the fields of cell culture, biomedical engineering and molecular biology will lead to more rapid advances in this area.

Cryopreservation of ovarian tissue has been reported to delay the development of preantral follicles during *in vitro *culture. Choi *et al*. [67] studied the effects of cryopreservation on the proliferation of granulosa cells during culture of mouse preantral follicles to understand the underlying mechanism of delayed follicular development. He found a significant reduction in granulosa cell proliferation of the cryopreserved preantral follicles compared to that of the fresh controls. Moreover, expressions of cyclin D2, Cdk4, cyclin E and Cdk2 also were reduced in the vitrified ovarian tissues at 0 and 24 h after culture. It is apparent from this study that the granulosa cell death and temporary suppression of granulosa cell proliferation through cell cycle regulators might account for delay in preantral follicle development following cryopreservation regimes. Current investigations show that the viability of caprine preantral follicles can be preserved best after solid surface vitrification in a mixture of sucrose and ethylene glycol followed by washes in medium containing sucrose, as opposed to the conventional vitrification procedure [68].

Choi *et al*. [69] evaluated the effect of cryopreservation (slow freezing and vitrification) on the development of frozen-thawed mouse primordial follicles. A significant reduction was noted in the developmental rate after *in vitro *culture of primordial follicles derived from both types of freezing methods, with a slight reduction in the mRNA expression (GDF-9, inhibin-alpha subunit and ZP3) levels than the fresh controls after five days of *in vitro *culture. Continuous agitation while culturing frozen-thawed ovarian issues have been shown to reduce degeneration of follicles [70].

## In vitro maturation of oocytes from antral follicles

Currently more than 300 healthy infants have been born following immature oocyte retrieval and IVM. Follow-up studies have reported no major concerns about the pregnancies, deliveries or health of the babies. Collection of small antral follicles with *in vitro *oocyte maturation is entering the mainstream of assisted reproductive technologies and is considered a better option for patients having polycystic ovarian syndrome (PCOS).

PCOS is characterized by abnormal endocrine parameters, anovulation, sonograms showing multiple antral follicles within the ovarian cortex and, frequently, infertility. Priming of ovarian immature oocytes with follicle-stimulating hormone or human chorionic gonadotropin prior to immature oocyte retrieval has been shown to improve oocyte maturation rates and embryo quality as well as pregnancy rates in these patients. Several factors determine the successful outcome with IVM of antral follicles. The size of follicles may be an important factor for subsequent embryonic development, but the developmental competence of oocytes originating from the small antral follicles is not adversely affected by the presence of a dominant follicle. Oocyte maturation (nuclear as well as cytoplasmic) also is affected profoundly by culture conditions. In general, the clinical pregnancy and implantation rates have reached 30–35% and 10–15%, respectively in infertile women with PCO or PCOS [71]. However, a recent report by Buckett *et al*. [72], which showed a higher rate of clinical miscarriage after IVM compared with IVF and intracytoplasmic sperm injection, warrants more rigorous, controlled studies on the efficacy of IVM, especially in PCOS. The possible long-term effects of IVM on the health and development of children also needs further study. At this juncture, it is imperative to say that couples should be counseled carefully about the current uncertainties involved in the assisted conception modalities used.

## Conclusion

The literature shows a substantial body of relevant works in organ cryopreservation, as reviewed by Fahy *et al*. [73], and this may pave the way for a successful development of cryopreservation regimens for ovarian tissue by vitrification. The ability to consistently recover rabbit kidneys after cooling to core temperatures of about -45°C with subsequent long-term life support function after transplantation constitutes a major achievement and gives the strongest evidence to date that the vitrification of whole organs may be achievable [73]. Physical studies seem indispensable for the progress of organ cryopreservation by vitrification [74].

As ovarian tissue cryopreservation enters the mainstream of assisted reproductive technology, the study of follicle biology and the search for an ideal *in vitro *follicle culture system are of major interest. One main concern with autografting of frozen-thawed ovarian grafts from cancer patients is the risk of re-introducing cancer cells. In this regard, frozen-thawed follicle culture is a more attractive goal in the long term because it would eliminate any risk of re-implanting any residual cancer cells and could in theory, produce more mature oocytes by avoiding follicle wastage created by ischemia or normal atresia. Despite the fact that follicle culture techniques have improved significantly due to ongoing active research, concerns about the formation and integrity of imprints in oocytes growing and ripening *in vitro *remain because defects could manifest as embryonic death or unhealthy offspring. The premature death and abnormalities of the first mouse ever produced from a primordial follicle *in vitro *serves as a warning and challenge for cryobiologists [46].

In conclusion, research should focus on refining cryopreservation and transplantation protocols to prevent the destructive effect of ischemic injury on follicular viability. The use of vascularized grafts and selection of an appropriate transplantation site may offer a plausible solution.

## Competing interests

To the authors' best knowledge, no competing interests of any nature arise from the current publication.

## Authors' contributions

ACV was responsible for the conceptualization and preparation of the manuscript. SSP and AA participated in preparation of the manuscript, while SSP performed the proofreading and final production. All figures were prepared by ACV, but the final content was discussed and reviewed by all four authors.
